# Coevolution between Stop Codon Usage and Release Factors in Bacterial Species

**DOI:** 10.1093/molbev/msw107

**Published:** 2016-06-13

**Authors:** Yulong Wei, Juan Wang, Xuhua Xia

**Affiliations:** ^1^Department of Biology, University of Ottawa, Ottawa, ON, Canada; ^2^Ottawa Institute of Systems Biology, Ottawa, ON, Canada

**Keywords:** translation termination, release factors, stop codon, RF1, RF2, *prfA*, *prfB*, gene expression.

## Abstract

Three stop codons in bacteria represent different translation termination signals, and their usage is expected to depend on their differences in translation termination efficiency, mutation bias, and relative abundance of release factors (RF1 decoding UAA and UAG, and RF2 decoding UAA and UGA). In 14 bacterial species (covering Proteobacteria, Firmicutes, Cyanobacteria, Actinobacteria and Spirochetes) with cellular RF1 and RF2 quantified, UAA is consistently over-represented in highly expressed genes (HEGs) relative to lowly expressed genes (LEGs), whereas UGA usage is the opposite even in species where RF2 is far more abundant than RF1. UGA usage relative to UAG increases significantly with P_RF2_ [=RF2/(RF1 + RF2)] as expected from adaptation between stop codons and their decoders. P_RF2_ is > 0.5 over a wide range of AT content (measured by P_AT3_ as the proportion of AT at third codon sites), but decreases rapidly toward zero at the high range of P_AT3_. This explains why bacterial lineages with high P_AT3_ often have UGA reassigned because of low RF2. There is no indication that UAG is a minor stop codon in bacteria as claimed in a recent publication. The claim is invalid because of the failure to apply the two key criteria in identifying a minor codon: (1) it is least preferred by HEGs (or most preferred by LEGs) and (2) it corresponds to the least abundant decoder. Our results suggest a more plausible explanation for why UAA usage increases, and UGA usage decreases, with P_AT3_, but UAG usage remains low over the entire P_AT3_ range.

## Introduction

Most bacterial lineages share genetic code 11 with three stop codons, UAA, UAG, and UGA, which are decoded by two release factors (RF1 and RF2), with RF1 decoding UAA and UAG and RF2 decoding UAA and UGA (Scolnick et al. 1968; Milman et al. 1969; Scolnick and Caskey 1969). In *Escherichia coli*, RF2 is consistently more abundant then RF1, which is associated with UGA used much more frequently than UAG. This association between the frequency of stop codon and its decoder concentration is consistent with codon–anticodon adaptation documented in bacteria (Ikemura 1981, 1992; Gouy and Gautier 1982; Xia 1998; Stoletzki and Eyre-Walker 2007; Higgs and Ran 2008; Palidwor et al. 2010; Ran and Higgs 2012), eukaryotes (Chavancy et al. 1979) such as yeast (Sharp and Li 1986; Sharp et al. 1986; Xia 1998; Akashi 2003) and fruit flies (Moriyama and Hartl 1993; Akashi 1994, 1997; Moriyama and Powell 1997), viruses (Sharp et al. 1984; van Weringh et al. 2011; Chithambaram et al. 2014a, 2014b; Prabhakaran et al. 2014, 2015), and mitochondria (Xia 2005, 2008; Xia et al. 2007; Carullo and Xia 2008; Jia and Higgs 2008).

Because different stop codons may manifest as different signals to the cellular translation termination machinery, both experimental and bioinformatic approaches have been taken to characterize translation termination efficiency in association with their decoders. The experimental studies on translation termination have focused mainly on *E. coli* (and occasionally in the yeast, *Saccharomyces cerevisiae*) and addressed two questions: (1) which tRNA species tend to misread a stop codon as a near-cognate sense codon and (2) which release factor tends to misread near-cognate sense codons as stop codons.

All three stop codons can be misread by tRNAs, and UGA appears to be the leakiest of the three, with a readthrough frequency of at least 10 ^−^ ^2^–10 ^−^ ^3^ in *Salmonella typhimurium* (Roth 1970) and *E. coli* (Sambrook et al. 1967; Strigini and Brickman 1973). UAA and UAG can also be leaky in bacteria (Davies et al. 1966; Ryden and Isaksson 1984), although their misreading has not been reported as frequently as UGA. Natural UAG readthrough frequency is mostly within the range of 1.1×10 ^−^ ^4^–7×10 ^−^ ^3^, depending on the nature of the downstream nucleotides (Bossi and Ruth 1980; Bossi 1983; Miller and Albertini 1983; Ryden and Isaksson 1984). The readthrough of UAA seems to occur at frequencies from 9×10 ^−^ ^4^ to < 1×10 ^−^ ^5^ (Ryden and Isaksson 1984). Overall, the available experimental data suggest that in bacteria species, particularly in *E. coli*, readthrough is most frequent for UGA, less for UAG, and least for UAA (Strigini and Brickman 1973; Geller and Rich 1980; Parker 1989; Jorgensen et al. 1993; Meng et al. 1995; Cesar Sanchez et al. 1998; Tate et al. 1999).

Translation termination error rate depends not only on readthrough by tRNA, but also on the efficiency and relative concentration of RF1 and RF2 (Korkmaz et al. 2014). Increasing RF2 concentration decreased both UGA readthrough and frameshift (reviewed in Tate et al. 1999). The observation that UAA is the most frequently used stop codon in *E. coli*, *Bacillus subtilis*, and *S. cerevisiae* (Sharp and Bulmer 1988) was interpreted in light of the fact that UAA has the largest number of decoders (being decoded by both RF1 and RF2) and that it is the most reliable stop signal of the three as reviewed above. Early studies suggest that RF1 and RF2, given the same concentration, decode their respective stop codons with roughly equal efficiency (Scolnick et al. 1968; Jorgensen et al. 1993; Freistroffer et al. 2000; Ito et al. 2000), and that both are extremely efficient and accurate against near-cognate codons, except for UGG in the case of RF2 and UAU in the case of RF1 (Freistroffer et al. 2000). However, given the same codon context, RF2 decoding UGA is less efficient than RF1 decoding UAG in *E. coli* (Bjornsson and Isaksson 1996).

The effect of both mutation and selection (mediated by relative concentration of RF1 and RF2) on stop codon usage have been studied. The selection effect is derived as an extension of the well-known codon–anticodon adaptation (Ikemura 1981, 1992; Akashi and Eyre-Walker 1998; Xia 1998; van Weringh et al. 2011; Chithambaram et al. 2014a, 2014b; Prabhakaran et al. 2014, 2015). As UGA is decoded only by RF2 and UAG only by RF1, one expects UGA to be used more than UAG when RF2 concentration is higher than RF1 (assuming that the two have equal decoding efficiency on their respective codons). This is consistent in *E. coli*, where RF2 is ∼5 times more frequent than RF1 (Adamski et al. 1994; Mora et al. 2007) and UGA is used much more frequently than UAG.

The mutation effect on stop codon usage is mainly studied through genomic GC content which has a strong effect on stop codon usage based on data from 736 species (Povolotskaya et al. 2012). An even more comprehensive compilation involving 4,684 genomes (Korkmaz et al. 2014) have revealed strong effect of GC content on the frequencies of UAA and UGA, but little on the frequency of UAG. However, the effect of GC content on stop codon usage depends on gene expression (Korkmaz et al. 2014).

These bioinformatic studies (Sharp and Bulmer 1988; Brown et al. 1993; Cridge et al. 2006; Povolotskaya et al. 2012; Korkmaz et al. 2014) have generally found UAA to be the most frequent stop codon and UAG the least frequent. In particular, Korkmaz et al. (2014) claimed that “TAG is truly a minor stop codon in all aspects”. Designating codons as major and minor codons are important not only in understanding the function of the translation machinery, but also in biopharmaceutical industry as many experimental studies have shown that replacing minor codons by major codons increases protein production (Robinson et al. 1984; Sorensen et al. 1989; Haas et al. 1996; Ngumbela et al. 2008).

The term “major (or minor) codon” is often misunderstood. “Major codon” (or optimal codon) originally refers to sense codons preferred by highly expressed genes and decoded by the most abundant tRNA. It is first used by McPherson (1988) in reference to a study (Kurland 1987) showing that highly expressed genes use codons to optimize decoding efficiency of the tRNA pool. A minor codon is the opposite. Major and minor codons are not necessarily the most frequent or least frequent codons when compilation is done for all genes.

Two criteria, one essential and one corroborative, have been used, sometimes implicitly, to identify a minor sense codon. The essential criterion is that a minor codon is the most strongly avoided in highly expressed genes (HEGs, in contrast to lowly expressed genes or LEGs). The corroborative criterion is that a minor codon corresponds to the least abundant tRNA among synonymous codons. Without these two criteria, a minor codon could be identified incorrectly (Xia 2015). For example, if we compile the codon frequencies of Asp codon family for all genes in *E. coli* (NC_000913), we will get 41,806 GAU and 25,015 GAC, which would mislead us to conclude that GAU is the major codon, and GAC the minor. However, if we rank *E. coli* genes by the protein abundance data compiled in the integrated data set in PaxDB (Wang et al. 2012) or by the index of translation elongation (I_TE_, Xia 2015), then LEGs (100 genes at the low end of abundant proteins) uses more GAU than GAC, but HEGs (100 genes at the high end of gene expression) uses more GAC than GAU. Furthermore, these Asp codons are translated by three tRNA^Asp^ genes all with the same GUC anticodon forming perfect base-pair with GAC. Thus, both criteria support GAC as the major (optimal) codon, and GAU as the minor.

Korkmaz et al. (2014) made an effort to apply these two criteria in identifying major and minor stop codons in bacteria. They compiled 4,684 bacterial genomes and concluded that “in all these phyla, TAG is the minor stop codon”, and that “TAG is truly a minor stop codon in all aspects”. The conclusion, however, is wrong because of misapplication of the two criteria, which may be best illustrated by taking *Microcystis aeruginosa* for example. LEGs use more UGA than UAG as stop codons in this species (P_UGA,LEG _=_ _0.2970, P_UAG.LEG_ = 0.2393, table 1), but HEGs use more UAG than UGA (P_UGA,HEG _=_ _0.2536, P_UAG.HEG_ = 0.1556, table 1). This stop codon usage pattern is consistent with the relative RF1 and RF2 concentrations compiled in the integrated data set available in PaxDB (Wang et al. 2012). Protein abundance is 33.3 ppm (parts per million) for RF1 and 18.2 ppm for RF2 in that integrated data set. The average concentration of RF1 is also higher than RF2 based on multiple separate measurements (table 1). Thus, UAG has more decoders than UGA and is expected to be more preferred than UGA by HEGs, especially given the experimental evidence (reviewed above) that UAG is a more accurate stop signal than UGA. So UAG clearly is not a minor stop codon in *M. aeruginosa*, contrary to what Korkmaz et al. have claimed. Korkmaz et al. (2014) used ribosomal protein and translation factor genes (which are generally highly expressed) as HEGs in a subset of genomes studied, but they did not contrast between HEGs and LEGs, so one does not know the difference in relative stop codon preference between HEGs and LEGs.

**Table 1:** Bacterial Species with Both RF1 and RF2 Concentrations (in ppm, with mean values presented for multiple measurements) in PaxDB (Wang et al. 2012), Together with Stop Codon Usage in Highly Expressed and Lowly Expressed Genes (HEGs and LEGs).

Species^a^	*N*_gene_^b^	RF1	RF2	P_AT3_^c^	P_RF2_^d^	P_UAA.LEG_^e^	P_UAA.HEG_	P_UAG.LEG_	P_UAG.HEG_	P_UGA.LEG_	P_UGA.HEG_
*E. coli*	1,000	53.1	453.00	0.4383	0.8951	0.5730	0.7770	0.1070	0.0320	0.3200	0.1910
*Y. pestis*	300	11.6	672.00	0.4979	0.9830	0.6100	0.7433	0.1300	0.0700	0.2600	0.1867
*M. tuberculosis*	800	200.5	548.50	0.2018	0.7323	0.1525	0.1688	0.2713	0.3538	0.5763	0.4775
*S. enteric*	600	59.2	142.89	0.4008	0.7070	0.5717	0.7650	0.1083	0.0433	0.3200	0.1917
*L. lactis*	300	45.5	98.05	0.7247	0.6833	0.7167	0.9100	0.1067	0.0467	0.1767	0.0433
*P. aeruginosa*	500	56.4	167.00	0.1262	0.7475	0.0560	0.2640	0.1280	0.0480	0.8160	0.6880
*H. pylori*	300	157.0	214.00	0.5777	0.5768	0.6267	0.6600	0.1567	0.1567	0.2167	0.1833
*L. interrogans*	600	139.3	183.00	0.6969	0.5677	0.5683	0.6467	0.1317	0.0983	0.3000	0.2550
*M. aeruginosa*	1,000	35.1	27.00	0.6059	0.4348	0.4639	0.5908	0.2392	0.2536	0.2970	0.1556
*S. pyogenes*	301	246.5	74.65	0.6766	0.2324	0.5748	0.7902	0.2292	0.1475	0.1960	0.0623
*B. subtilis*	1,000	216.0	205.00	0.5518	0.4869	0.5600	0.7300	0.1530	0.1240	0.2870	0.1460
*B. anthracis*	300	94.3	4.59	0.7349	0.0464	0.7367	0.8567	0.1367	0.0867	0.1267	0.0567
*S. aureus*	392	496.0	47.70	0.7702	0.0877	0.7398	0.8475	0.1633	0.1025	0.0969	0.0500
*A. ferrooxidans*	301	425.5	377.00	0.3096	0.4698	0.2425	0.3033	0.1362	0.1433	0.6213	0.5533

^a^Full species names are, in the same order, *Escherichia coli*, *Yersinia pestis* CO92, *Mycobacterium tuberculosis*, *Salmonella enterica*, *Lactococcus lactis*, *Pseudomonas aeruginosa*, *Helicobacter pylori*, *Leptospira interrogans*, *Microcystis aeruginosa*, *Streptococcus pyogenes*, *Bacillus subtilis*, *Bacillus anthracis*, *Staphylococcus aureus* sp. Mu50, and *Acidithiobacillus ferrooxidans*.

^b^Number of genes in top and bottom 25% on the gene expression scale (ranked by either protein abundance values in PaxDB). If 25% includes >1,000 genes, then use 1,000.

^c^Proportion of AT at third codon site.

^d^Proportion of RF2, i.e., RF2/(RF1 + RF2).

^e^Proportion of UAA stop codons in LEGs. The same format applies to the last five columns.

For relative abundance of RF1 and RF2, Korkmaz et al. (2014) only confirmed previous findings that RF2 is several fold more abundant than RF1 in *E. coli*, but did not have RF1 and RF2 abundance data for the rest of the 4,684 species they studied. For the two other species that they studied in detail, *B. subtilis* and *Mycobacterium smegmatis*, they have only mRNA data for *prfA* (coding RF1) and *prfB* (coding RF2). However, more *prfB* mRNA than *prfA* mRNA does not imply more RF2 than RF1 because RF2 is translationally regulated (Craigen et al. 1985; Donly et al. 1990). Thus, their key conclusion that “UAG is truly a minor stop codon in all aspects” is an unwarranted generalization.

Korkmaz et al. (2014) did notice that UAG in some bacterial species is more frequent than UGA. However, they interpreted these observations as likely arising from the process of UGA reassignment to a sense codon. They in particular drew attention to Mollicutes where many lineages use genetic code 4 with only two stop codons (UAA and UAG, with UGA reassigned to tryptophan). However, their Table 2 included bacterial species where UAG is used frequently, with no evidence that UGA is either reassigned or in the process of being reassigned. Korkmaz et al. also speculated that the combination of UAG and RF1 is translationally less efficient and accurate than that of UGA and RF2 which, however, is contrary to available experimental evidence reviewed above.

It may be entirely unnecessary to argue that UAG is a nearly universal minor stop codon in bacteria. Those bacterial species that use more UAG than UGA as stop codons may not at all be in the process of having UGA reassigned to sense codons, but instead may simply have more actively decoding RF1 than RF2 in their cells. This hypothesis, which may be termed codon–decoder adaptation hypothesis, is consistent with many previous experimental and bioinformatic studies, including Korkmaz et al. (2014). In fact, one of the key contributions in Korkmaz et al. (2014) is the confirmation that stop codon usage in *E. coli* is related to relative abundances of RF1 and RF2.

Proteomic studies have been carried out in many bacterial species, with 14 of them (covering Proteobacteria, Firmicutes, Cyanobacteria, Actinobacteria and Spirochetes) having both RF1 and RF2 quantified and deposited in PaxDB (Wang et al. 2012). Of particular value in these data is that relative abundance of RF1 and RF2 varies widely, which paves the way for evaluating the effect of relative abundance of RF1 and RF2 on stop codon usage. The availability of protein abundance data for thousands of proteins also permits a more objective and comprehensive characterization of HEGs and LEGs and their respective stop codon usage.

We found UAA consistently over-represented in HEGs relative to LEGs, consistent with experimental studies (reviewed above) showing UAA to be the most efficient stop codon. In contrast, UGA is always avoided in HEGs relative to LEGs. This is true even in species where UGA accounts for an overwhelming majority of stop codons and RF2 is far more abundant than RF1. In such species, UAA is mostly found in HEGs. UGA usage relative to UAG increases significantly with relative abundance of RF2, following the expectation that synonymous codons increase in usage with the abundance of their decoders (which are tRNAs in the case of sense codons and release factors in the case of stop codons). RF2 is more abundant than RF1 over a wide range of AT content, but decreases rapidly toward zero at extreme AT-richness. This explains why bacterial lineages with high genomic AT content often have UGA reassigned because the low RF2 would select strongly against UGA. There is no indication that UAG is a minor stop codon in bacteria as claimed by Korkmaz et al. (2014). Our results suggest a more plausible explanation for why UAA usage increases, and UGA usage decreases, with P_AT3_, but UAG usage remains low over the entire P_AT3_ range.

## Results and Discussion

We ranked protein-coding genes by the following: (1) protein abundance and (2) index of translation efficiency (I_TE_), and the top 25% and bottom 25% of genes are taken as HEGs and LEGs (see Materials and Methods for details). We defined P_UAA_, P_UGA_ and P_UAG_ as the proportion of the three stop codons, and P2_UGA _=_ _N_UGA_/(N_UGA _+_ _N_UAG_), where N_UGA_ and N_UAG_ are the number of UGA and UAG codons. Note that P2_UGA_ is different from P_UGA_ which is N_UGA_/(N_UGA _+_ _N_UAA _+_ _N_UAG_). P_UAA_, P_UGA_, P_UAG_ and P2_UGA_ based on HEGs or LEGs will be subscripted by “HEG” or “LEG”, respectively. We also defined P_RF2_ as [RF2]/([RF1] + [RF2]), where [X] is the concentration of X. We used AT content at the third codon site (P_AT3_) as a proxy of AT-biased mutation.

To facilitate presentation, we rebranded the conventional codon–anticodon adaptation hypothesis for sense codons as codon–decoder adaptation hypothesis. This generalized hypothesis predicts that a codon, be it sense or stop codon, increases its usage with its decoders, and that such increase is typically more pronounced in HEGs than in LEGs.

### UAA is a Major Codon in All 14 Species

**Fig. 1.**
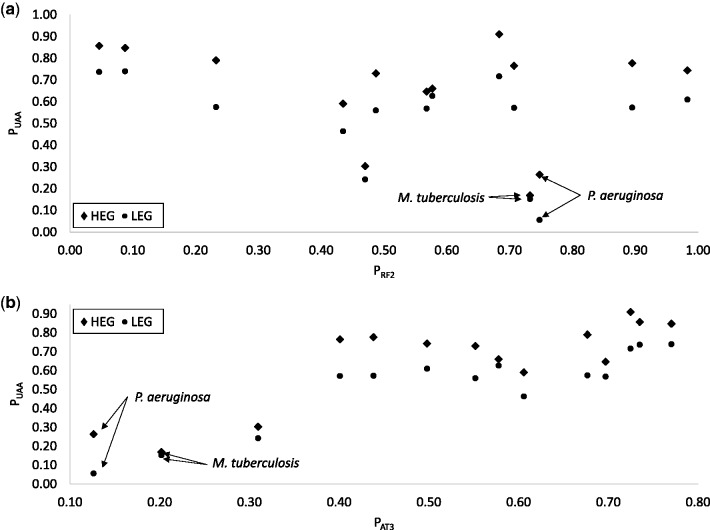
Stop codon UAA is preferred in highly expressed genes (HEGs) relative to lowly expression genes (LEGs) in all 14 species, regardless of (*a*) relative abundance in RF1 and RF2, measured by PRF2 as RF2/(RF1 + RF2), or (*b*) proportion of AT at third codon site (P_AT3_).

**Fig. 2.**
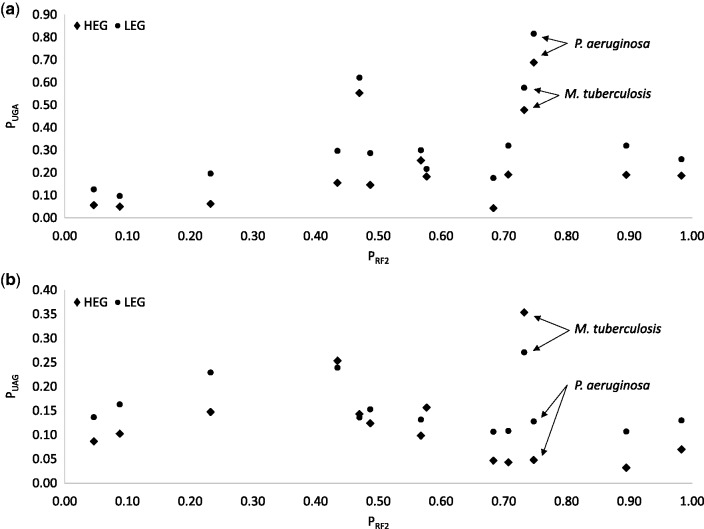
Stop codon UGA is never preferred in HEGs relative to LEGs even RF2 is far more abundant than RF1 (*a*), and stop codon UAG is preferred in HEGs in 3 of the 14 species (*b*).

P_UAA_ does not increase or decrease with the relative availability of RF2 (P_RF2_, fig. 1a and table 1) which is expected because RF1 and RF2 can both decode UAA with roughly equal efficiency, at least in *E. coli* (Scolnick et al. 1968; Jorgensen et al. 1993; Freistroffer et al. 2000; Ito et al. 2000). What is remarkable is that P_UAA_ is always higher in HEGs than in LEGs in all 14 species (fig. 1), even in extremely GC-biased genomes (fig. 1b, P_AT3_ is only 0.1262 for *P. aeruginosa* and 0.2018 for *Mycobacterium tuberculosis*). In contrast, UGA is always avoided in HEGs relative to LEGs (fig. 1), even in species where UGA represents an overwhelming majority of stop codons in all genes. Among the 5,925 annotated protein-coding genes in *P. aeruginosa* (NC_011770), 4,651 terminate with UGA, 684 with UAG and only 590 with UAA (which are mostly in HEGs). This preponderance of UGA stop codons is associated with greater abundance of RF2 than RF1 (P_RF2 _=_ _0.7475 in *P. aeruginosa*). Given so many UGAs and so few UAAs in *P. aeruginosa*, one would have expected RF2 to evolve a higher efficiency to decode UGA, perhaps at the cost of reduced efficiency of decoding UAA, so that HEGs would have an increased preference for UGA relative to UAA. However, this expectation is not supported as UGA is used less frequently in HEGs than in LEGs in these two species (fig. 2a, P_UGA.HEG_ = 0.6880 and P_UGA.LEG_ = 0.8160 for *P. aeruginosa*). Thus, although UAA is rare in *P. aeruginosa*, it is strongly preferred by HEGs. In contrast, UGA in *P. aeruginosa* is frequent (and RF2 more abundant than RF1), yet it is avoided by HEGs. The difference in stop codon usage between 500 HEGs and 500 LEGs is highly significant based on chi-squared test with Yates correction for continuity (χ^2 ^=^ ^91.23, DF = 2, *P* < 0.0001). One possible explanation for this lack of expected RF2 evolution is that genomic AT content could change very quickly (Marin and Xia 2008; Nikbakht et al. 2014), whereas functional modification of a key cellular protein is typically a very slow process. In short, GC-biased mutation can increase UGA at the cost of UAA, but does not change the preference of UAA by HEGs in all 14 species studied.

In model organisms such as *E. coli*, UAA has been shown to be the most efficiently decoded and UGA the least (Strigini and Brickman 1973; Geller and Rich 1980; Parker 1989; Jorgensen et al. 1993; Tate et al. 1999). Highly expressed genes in *E. coli* were previously observed to prefer UAA as stop codons (Jin et al. 2002). Our result, with 14 species covering a wide taxonomic spectrum, suggests that UAA is a more efficient stop signal than other stop codons in bacteria in general. This implies that a transgenic gene expressed in a bacterial species should be terminated with UAA to enhance termination efficiency.

The other AT-poor species, *M. tuberculosis*, also exhibit strong difference between HEGs and LEGs (χ^2 ^=^ ^16.23, DF = 2, *P* = 0.0003), but here both UAG (fig. 2) and UAA (fig. 1) are preferred in HEGs relative to LEGs. The strong preference of UAG in HEGs is clearly at odds with the conclusion in Korkmaz et al. (2014) that “UAG is a minor stop codon in all aspects”. P_UAG.HEG_ is also higher than P_UAG.LEG_ in *M. aeruginosa* and *Acidithiobacillus ferrooxidans*, and the two are equal in *Helicobacter pylori* (table 1). Thus, UAA is universally preferred in HEGs, UAG is preferred in HEGs in 3 species, and UGA is avoided in HEGs in all 14 species.

If we do not contrast between HEGs and LEGs, and focus on HEGs only or all genes, then we may arrive at a wrong conclusion that UGA is the major codon and UAA the minor codon in *M. tuberculosis* and *P. aeruginosa* because UGA is more frequent than UAA or UAG. Take HEGs in *M. tuberculosis* for example. P_UGA.HEG_, P_UAG.HEG_ and P_UAA.HEG_ are 0.4775, 0.35375 and 0.16875, respectively (table 1). However, UGA is not the major codon because UGA is even more frequent than UAA or UAG in LEGs, with P_UGA.LEG_, P_UAG.LEG_ and P_UAA.LEG_ being 0.57625, 0.27125 and 0.1525, respectively (table 1). It is crucially important to contrast codon usage between HEGs and LEGs in identifying codons favoured by decoder-mediated selection (Eyre-Walker and Bulmer 1995; Xia 2015).

### Relative Usage of UAG and UGA Depends on Relative Abundance of RF1 and RF2

Because UAG is decoded by RF1 and UGA by RF2, we expect P2_UGA_, which is the proportion of UGA within (UGA + UAG), to increase with P_RF2_. The concentration of RF1 and RF2 vary widely among the 14 bacterial species, with P_RF2_ varying from 0.046 in *Bacillus anthracis* to 0.9830 in *Yersinia pestis* CO92. The codon–decoder adaptation hypothesis predicts that species like *B. anthracis* should use UAG more frequently than UGA in HEGs and species like *Y. pestis* CO92 should use UGA more frequently than UAG. We tested this prediction by using regression on the original P_RF2_ and P2_UGA_ and on phylogeny-based independent contrasts (Felsenstein 1985). The latter method alleviates the problem of data dependence due to sharing of ancestry.

**Fig. 3.**
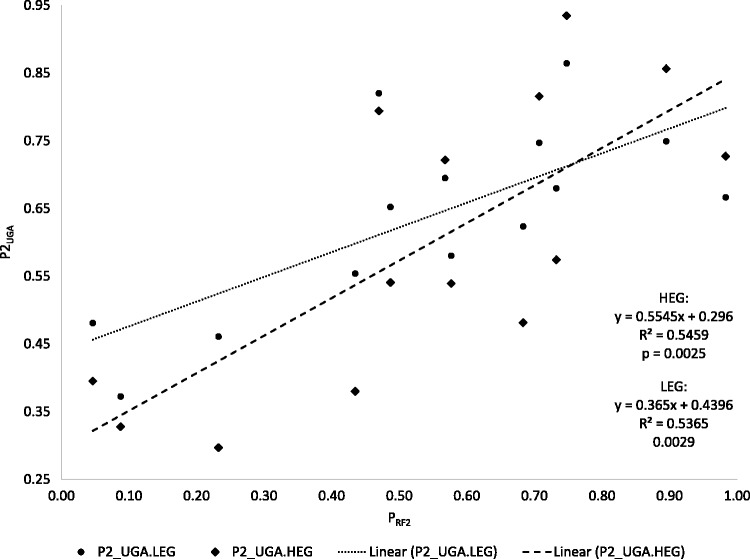
Relative usage of UGA and UAG, measured as P2_UGA _=_ _UGA/(UGA + UAG), increases significantly with relative abundance of RF2, measured as P_RF2_ = RF2/(RF1 + RF2).

The stop codon usage among the 14 bacterial species is as predicted by the codon–decoder adaptation hypothesis (fig. 3). First, both LEGs and HEGs follow the same trend with P2_UGA_ increasing with P_RF2_ (*P* < 0.01 in both LEGs and HEGs, fig. 3). Second, the pattern is stronger in HEGs than in LEGs. For example, in the three species with the highest P_RF2_ values, P2_UGA.HEG_ is greater than P2_UGA.LEG_ (fig. 3). In the three species with the lowest P_RF2_ values, P2_UGA.HEG_ is lower than P2_UGA.LEG_ (fig. 3). Such a pattern is consistent with that observed in sense codons. There is no indication that “UAG is truly a minor stop codon in all aspects” (Korkmaz et al. 2014), and there is consequently no need to invoke the speculations by Korkmaz et al. (2014) that the combination of UAG and RF1 is worse than that of UGA and RF2 in translation termination efficiency and accuracy. A codon becomes rare when its decoder is rare and vice versa. One may say that UAG is a minor codon in *E. coli*, but it is inappropriate to say that UAG is a universal minor codon and jump to speculate that the combination of UAG and RF1 is inefficient or inaccurate.

Based on the regression line for P2_UGA.HEG_ on P_RF2_, P2_UGA.HEG_ equals 0.5 when P_RF2 _=_ _0.3679 (i.e., when RF2:RF1 is ∼0.6:1). Thus, if we may make a liberal interpretation of this result from a limited data of 14 species, then UGA will tend to be less frequent than UAG (i.e., P2_UGA.HEG_ < 0.5) when P_RF2_ is < 0.3679, but UAG will tend to be less frequent than UGA when P_RF2_ is > 0.3679 (assuming equal efficiency between RF1 decoding UAG and RF2 decoding UGA). In our study, 3 of the 14 species (*Streptococcus pyogenes*, *B. anthracis*, and *Staphylococcus aureus*) have P_RF2_ <0.3679 (fig. 3) and their UGA, instead of UAG, is the less frequent of the two, with their P2_UGA.HEG_ values being 0.2969, 0.3953, and 0.3279, respectively. It is unnecessary to suggest, as Korkmaz et al. (2014) did, that bacterial species with low UGA usage may be in the process of UGA reassignment.

**Fig. 4.**
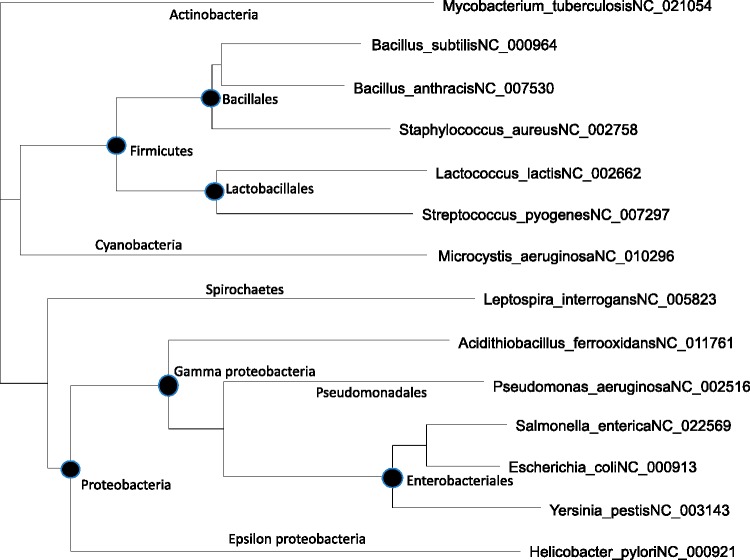
Phylogenetic tree built with small subunit ribosomal RNA sequences (ssu rRNA), used for independent contrasts, with leaves denoted by species name and GenBank accession for genomes from which the ssu rRNA sequences are extracted. Only the first annotated ssu rRNA sequence is used.

Strictly speaking, the regression and significance tests of the regression slope in figure 3 are not valid because the P2_UGA_ and P_RF2_ values are not independent due to the sharing of ancestry among the bacterial species. For example, *E. coli*, *S. enterica*, and *Y. pestis* are closely related, so are *B. subtilis* and *B. anthracis.* In the extreme case when two species are identical, then the two associated data points should really be treated as just one data point. To alleviate this problem, we have built a tree from the small subunit ribosomal RNA from the 14 species (fig. 4) and computed the independent contrasts (Felsenstein 1985) for P_RF2_ and P2_UGA_ based on the tree and the data in table 1. The results for regressing P2_UGA.HEG_ on P_RF2_ are slope = 0.3062, *r* = 0.5693, *P* =0.0336, and those for regressing P2_UGA.LEG_ on P_RF2_ are slope = 0.2663, *r* = 0.5800, *P* = 0.0297. This result does not depend heavily on the tree in figure 4. We have generated 100 bootstrap trees and repeated independent contrast analysis for each tree. The *P* value is always <0.05. Thus, P2_UGA_ depends significantly on P_RF2_, following the prediction of codon–decoder adaptation hypothesis.

### P_RF2_ Decreases with Genomic AT Bias

The wide variation in relative concentration of RF1 and RF2 (with P_RF2_ varies from 0.046 to 0.9830) raises the question of what affects P_RF2_. As previously noted (Korkmaz et al. 2014), bacterial species that lack the *prfB* gene and have UGA reassigned as a sense codon are typically associated with high genomic AT content. It is therefore reasonable to hypothesize that RF2 abundance decreases with AT content and disappears in species with extreme AT-bias so that UGA as a stop codon would be strongly selected against and eventually reassigned.

**Fig. 5.**
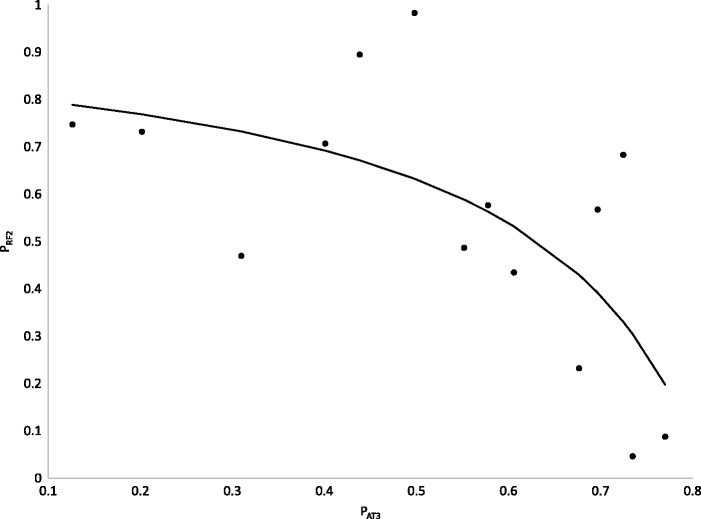
Relative abundance of RF2 decreases rapidly at high range of AT content, measured by proportion of AT at third codon site (P_AT3_).

AT bias, measured by either the third codon position or by inter-gene sequences, indeed is negatively and highly significantly related to P_RF2_ (fig. 5, the Spearman rank correlation is −0.6659, *P* = 0.0093, where P_AT3_ is the proportion of AT at third codon sites, and is similar to the proportion of AT in intergenic sequences). The relationship can be fitted well by the following equation:

(1) $$P_{RF2}=\frac{0.81566-P_{AT3}}{1-P_{AT3}}$$

The fitted curve (fig. 5), which accounts for 46.94% of the variation in P_RF2_, implies that P_RF2_ will rapidly approach 0 when P_AT3_ approaches 0.81566 or higher. This trend that P_RF2_ would approach 0 with increasing P_AT3_ explains why extremely AT-rich bacterial genomes frequently lose *prfB* and have stop codon UGA reassigned. The equation also explains why RF2 is more likely lost than RF1 because the concentration of RF1 does not approach 0 with changes in P_AT3_ (fig. 5). These results offer empirical substantiation of previous models on stop codon reassignment (Osawa and Jukes 1989, 1995; Andersson and Kurland 1991; Sengupta and Higgs 2005; Sengupta et al. 2007).

We have previously mentioned that P2_UGA.HEG_ tends to be < 0.5 (i.e., more UAG than UGA) when P_RF2_ is < 0.3679. According to equation (1), P_RF2_ will be < 0.3679 when P_AT3 _>_ _0.70835. This result, if interpreted liberally, suggests that UAG will tend to be more frequent than UGA only when P_AT3_ is >0.70835, and explains why UAG tends to be the least frequent in most bacterial species because relatively few bacterial genomes have P_AT3 _>_ _0.70835.

### Dynamic Changes of Stop Codons with AT Content

**Fig. 6.**
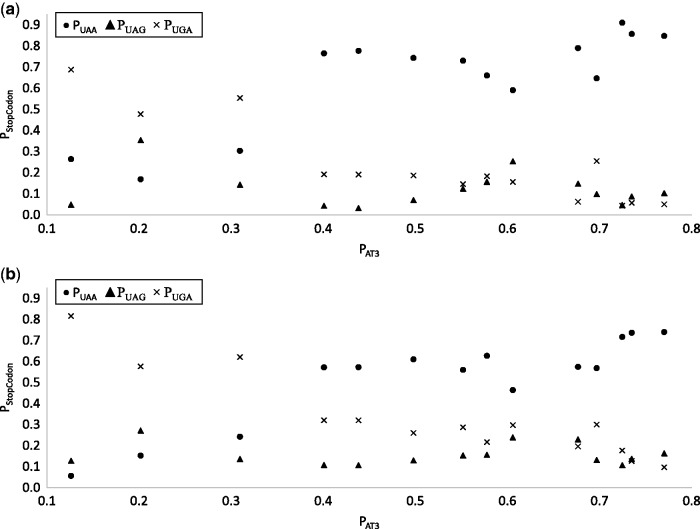
UAA usage increases, and UGA usage decreases, with P_AT3_, but UAG usage is low and changes little with P_AT3_. The pattern is consistent in both highly expressed genes (*a*) and lowly expressed genes (*b*).

One conspicuous pattern observed previously (Korkmaz et al. 2014) is that UAA usage increases, and UGA usage decreases, with AT content, but UAG usage remains low and hardly changes with AT content. This pattern is also visible in the 14 species here (fig. 6). Korkmaz et al. (2014) interpreted this pattern as consistent with UAG being a minor codon that has translation termination efficiency and accuracy problems and is therefore nearly universally avoided. This interpretation by Korkmaz et al. (2014) is somewhat far-fetched for two reasons. First, as we have mentioned earlier, experimental evidence suggests that UAG is typically more efficient and accurate than UGA as a termination signal. Second, UAG is favored by HEGs in 3 of the 14 species whereas UGA is avoided by HEGs in all 14 species. Furthermore, the interpretation does not explain why UGA becomes less frequent than UAG at high AT content which is particularly visible in Figure 2B in Korkmaz et al. (2014) for highly expressed genes.

Our results on the change of P_RF2_ and P_AT3_ offer an alternative explanation for the observation of the following: (1) low UAG usage and (2) little change in UAG usage over the entire range of AT content in bacterial genomes. At the low P_AT3_ range, mutation would have favoured both UAG and UGA at the cost of UAA. However, P_RF2_ is high with low P_AT3_ (fig. 5) which would favor UGA and select against UAG, keeping the latter at low frequency. At high P_AT3_, mutation would favor UAA against UGA and UAG and we expect the latter two to decrease. However, P_RF2_ approaches 0 at high P_AT3_ (fig. 5), which selects strongly against UGA codons, but little against UAG codons (as RF1 becomes the dominant release factor at high P_AT3_). This explains why, at high P_AT3_, UAG does not decrease as much as UGA in figure 6a and tend to have its usage higher than that of UGA. This pattern is also visible in Figure 2B in Korkmaz et al. (2014). In the mid-range of P_AT3_, UAA is overused because of the following: (1) it is favoured by selection and (2) there is no mutation bias against it. Also in this range, P_RF2_ is still much >0.5 (fig. 5), favoring UGA against UAG and keep the latter at low frequency. So UAG usage is kept low and changes little over the entire range of P_AT3_.

## Materials and Methods

### Classifying Genes According to Gene Expression

We have used protein abundance and Index of Elongation Efficiency (I_TE_, Xia 2015) as proxies of gene expression. Protein abundance data were downloaded from PaxDB (Wang et al. 2012). For species with multiple proteomic studies, only the integrated data set is downloaded and used to rank the coding sequences. The protein ID in PaxDB is often the Uniprot ID and needs to be mapped to gene names (or GI or GeneID) in a GenBank file for individual species (e.g., *B. subtilis*). We downloaded the paxdb-uniprot-links file relevant to the species (e.g., 224308-paxdb_uniprot.txt for *B. subtilis*), saved the Uniprot ID (the last column) to a file (e.g., BsUniprotID.txt), browsed to http://www.uniprot.org/uploadlists/ (last accessed May 31, 2016), under “Provide your identifiers” uploaded the BsUniprotID.txt file, under “Selection options” selected the mapping from “UniProtKB AC/ID” to “Gene name” (or GI or GeneID), and clicked “Go”. The resulting mapping file was generated with two columns (original input Uniprot IDs and the mapped gene name (or GIs GeneID) corresponding to gene name or other IDs in a GenBank file. Unmapped ID is stored in a separate file, also available for downloading.

An alternative proxy for gene expression is I_TE_ which require codon usage data from both HEGs and LEGs. For each species, we ranked the genes by protein abundance, took the top 40 ribosomal protein genes as HEGs and bottom 40 genes with nonzero values as LEGs, and compiled codon usage table for HEGs and LEGs separately. These codon usage tables were then used to compute I_TE_ with DAMBE. The resulting I_TE_ is then used as a proxy of gene expression. The advantage of using I_TE_ is that it can be used for all genes and that it is less affected by differential mRNA abundance and protein degradation.

After genes were ranked by either protein abundance or I_TE_, We have used top and bottom 25% of genes as HEGs and LEGs, respectively, to compile stop codon usage, so the actual number of genes taken as HEGs and LEGS differ among species. If 25% of genes is >1,000, then only 1,000 genes were used. The two ways of ranking genes by their expression (i.e., by protein abundance or by I_TE_) lead to similar results. The results presented are based on the ranking by protein abundance. The results from ranking by I_TE_ have slightly stronger patterns with slightly smaller *P* values.

### RF1 and RF2 Concentration

We compiled RF1 and RF2 concentration from proteomic data at PaxDB. Only 14 species have both RF1 and RF2 measured and were included. An average is used when multiple values available. Our values are therefore not always the same as those RF1 and RF2 values in the integrated data sets in PaxDB because the latter includes studies in which either RF1 or RF2 is measured.

### Phylogenetic Reconstruction

For computing phylogeny-based independent contrasts, we extracted small subunit ribosomal RNA (ssu rRNA) sequences from genomic sequences in GenBank (with accession included in fig. 4). For species with multiple ssu rRNA genes, only the first one is used for phylogenetic reconstruction. The sequences were aligned by MAFFT (Katoh et al. 2009) with the slow but accurate “–localpair” and “–maxiterate = 1,000” options.

Two phylogenetic reconstruction methods were used. The first was PhyML (Guindon and Gascuel 2003) with GTR (or HKY85). The tree improvement option “-s” was set to “BEST” (best of NNI and SPR search). The “-o” option (optimize starting tree) was set to “tlr” which optimizes the topology, the branch lengths and rate parameters. The other was a distance-based FastME method (Desper and Gascuel 2002, 2004) implemented in DAMBE (Xia 2013), with the simultaneously estimated maximum composite likelihood distance (Tamura et al. 2004; Xia 2009) based on the TN93 model (MLCompositeTN93). The two trees from the two methods have identical topology and almost perfectly correlated branch lengths. The independent contrasts were generated by using the CONTRAST program in the PHYLIP package (Felsenstein 2014).
